# Connectome Analysis of the Nucleus Accumbens: Refining Radiosurgical Targeting for Addictive Disorders

**DOI:** 10.7759/cureus.95307

**Published:** 2025-10-24

**Authors:** Jose E Chang, William O Contreras Lopez, Richard Gonzalo Párraga, Jorge Torres Monterrosa, Larissa Merlos Salazar, Eduardo E Lovo

**Affiliations:** 1 Neurosurgery-Gamma Knife Program, International Cancer Center, Diagnostic Hospital, San Salvador, SLV; 2 Functional Neurosurgery, International Neuromodulation Center (NEMOD) Clínica Foscal Internacional, Floridablanca, COL; 3 Functional Neurosurgery, Universidad Autónoma de Bucaramanga, Bucaramanga, COL; 4 Neurocirugía, Instituto de Neurocirugía Bolivia, Cochabamba, BOL; 5 General Practice, Universidad de El Salvador, San Salvador, SLV; 6 General Practice, Universidad Dr. José Matías Delgado, San Salvador, SLV

**Keywords:** addictions, connectomics, nucleus accumbens, radiomodulation, stereotactic radio surgery (srs)

## Abstract

Introduction: Addictions represent a major challenge not only for healthcare systems but also impose a significant socioeconomic burden affecting a large number of individuals worldwide. Various treatment modalities have been proposed, including pharmacological interventions, deep brain stimulation, and radiofrequency ablation, among others. Stereotactic radiosurgery has emerged as a non-invasive and precise alternative for addiction management. A thorough understanding of the nucleus accumbens (NAc) anatomy and its neural connections is essential to refine the radiosurgical target and optimize clinical outcomes.

Materials and methods: Diffusion tensor imaging was acquired from 3-Tesla and 1.5-Tesla Magnetic Resonance Imaging scanners on five healthy subjects, using T1 and T2 sequences. Images were fused, with autosegmentation of NAc, ventral tegmental area, amygdala, hippocampus, hypothalamus, and periventricular gray; insula, medial/lateral orbitofrontal cortex, and dorsolateral frontal cortex were manually drawn. Connectivity to NAc was assessed visually with fractional anisotropy thresholds (20-10). Five densest fiber connections were combined for target selection, aligned to the anterior and posterior commissure, and coordinates and structures were transferred to Gamma Plan for treatment planning.

Results: The strongest connections were found between the medial orbitofrontal cortex (mOFC), hypothalamus, ventral tegmental area (VTA), periventricular gray (PVG), and amygdala to the NAc. The mOFC-NAc and the hippocampus-NAc connections are the most and least robust, respectively. Stereotactic coordinates were derived for connectome-based targeting, proposing a 90 Gy dose to the NAc shell, predominantly, and aligning to it the 20 Gy isodose line for neuromodulation. The right and left NAc received > 20 Gy in 75.2% and 55.6% of their volumes, respectively. The optic apparatus received ≤ 5.4 Gy as Dmax.

Conclusion: Radiosurgery has proved to be a safe modality in the treatment of functional disorders and may offer a new treatment option for refractory addiction. Connectivity-based radiosurgery visualizes most of the NAc connections and may enhance patient-specific targeting.

## Introduction

The global burden of alcohol and drug addiction has gone well beyond individual health, putting pressure on healthcare sectors, economies, and social stability. Globally, a great amount of the population suffers from substance use disorders, including those caused by alcohol, opioids, and cannabis. The most common being alcohol use disorder (AUD), affecting 100.4 million worldwide. Followed by AUD are opioid dependence with 26.8 million cases and cannabis dependency with 22.1 million cases reported [1]. Global deaths attributable to substance use, reported in 2019, accounted for 583,511 across all drugs. Of this number, deaths directly attributable to drug use disorders alone were 181,758 (31.16%) [2].

Socioeconomic burden due to substance use is extremely high; for instance, the economic cost of opioid use disorder (OUD) and fatal opioid overdoses was estimated to be $1,020.7 billion in a thorough study conducted in 2017 [3]. Chronic exposure to addictive substances like the ones previously stated disrupts synaptic plasticity within the nucleus accumbens (NAc), playing a critical role in addiction by integrating dopaminergic, glutamatergic, and limbic inputs that mediate reward and craving. Its involvement in these processes has led to its exploration as a therapeutic target through pharmacological treatments, neuromodulation, and lesioning [4-6].

Stereotactic radiosurgery represents a promising non-invasive alternative; however, the precise definition of the optimal target within the ventral striatum remains a key challenge. Advances in diffusion tractography and connectomic methods now offer the possibility of refining this targeting to enhance efficacy and safety, while promoting individual patient anatomy-based radiosurgery as opposed to indirect targeting in neurosurgery.

Connectome-based radiosurgery is an emerging method that aims to further improve clinical efficacy through personalized targeting as opposed to indirect targeting, which has a more standard coordinate approach. In connectome-based targeting, as described by our group for pain [7], we utilize autosegmentation tools of a commercially available software to identify the area or target being treated and the maximum density of fibers crossing the target. Once this area has been identified, stereotactic coordinates can be obtained and then extrapolated to the Gamma Knife’s treatment planning station. Connectome-based targeting still has limitations, but it can be useful for refining target selection in radiosurgery treatments in the near future.

We aimed to characterize the most densely connected pathways to the NAc, from the cortical and subcortical areas involved in addiction neuronal circuitry, using fractional anisotropy (FA) and their correspondence to anatomical white matter tracts. Furthermore, we sought to identify the optimal connectomic-based target to provide a patient-specific treatment with radiosurgery.

## Materials and methods

The current study was approved by the International Cancer Center Ethics Committee under the number CECIC 2025/002; it did not involve any form of treatment or exposure of patients to any interventions. A three-tesla magnetic resonance imaging (MRI) Diffusion Tensor Imaging (DTI) study of a healthy subject, along with an axial 440-slice, 0.5 mm thickness three-dimensional (3D) Magnetization-Prepared Rapid Gradient Echo (MPRAGE) T1 sequence with and without contrast and an axial 3D T2 sequence with the same number of slices and characteristics of the entire head, was acquired using a Vantage Galan from Canon (Canon, Tochigi, Japan) and then transferred to BrainLab Elements (BrainLab, Munich, Germany). The same process, later described as connectomic, was performed using four different healthy subject studies with a 1.5-tesla machine, Siemens Avanto (Siemens, Erlangen, Germany), to validate connectivity findings in both systems. In the 1.5-tesla studies, only DTI and a 146-slice MPRAGE T1 sequence with 1 mm thickness and no spacing were acquired on this machine. Image fusion of the DTI study with the MPRAGE T1 sequence was carried out, and autosegmentation for the NAc, internal capsules, ventral tegmental area (VTA), amygdala (AMYG), hippocampus, hypothalamus, and periventricular gray (PVG) was performed; the areas that needed to be drawn were the insula, medial orbitofrontal cortex (mOFC), lateral orbitofrontal cortex (lOFC), and dorsolateral frontal cortex (dlFC) using Elements (BrainLab, Munich, Germany).

DTI-based connectomics of NAc

Different sequences of structures were “connected” to the NAc to obtain the various fiber connections; thickness was evaluated visually without modification of the FA. Often quantified as FA in diffusion tensor imaging (DTI) of the brain, FA is a crucial metric that reflects the directional dependence of water molecule diffusion within neural tissues, providing more information about the microstructural integrity of white matter tracts. In healthy brain tissue, water diffusion is highly anisotropic, resulting in high FA values (FA ≈ 1) that indicate well-organized fiber bundles. Conversely, reduced FA values (FA = 0, isotropic diffusion) can signal disruptions such as axonal damage, demyelination, other neurological conditions, or even healthy brain tissue such as gray matter or cerebrospinal fluid.

The cortical and subcortical structures connected to the NAC included the mOFC-lOFC-dlFC to NAc, the hypothalamus to NAc, the amygdala and hippocampus to NAc, the VTA to NAc, and, lastly, the PVG to NAc. No autosegmentation was available for medial thalamus structures; that being said, the PVG structure, with an added margin of 4 mm, was used as an anatomical representation of the medial thalamus structures to infer possible fiber connections between the NAc and medial thalamus. The connections of all structures were visually assessed with FA modifications ranging from 20 (the highest, default connection) to 10 (the lowest FA) to intensify weak connections, if any. Below an FA of 10, connections were considered non-existent if they did not appear, although the FA parameter could be lowered further. 

The five most densely connected fibers to the NAc were selected from a 1.5-tesla study and then simultaneously activated to visually create a single fiber bundle, which was used for target selection. Once the connections were established in the stereotactic module of the Elements program, the images were aligned to the anterior and posterior commissure (ACPC), and a target was selected for each hemisphere at the point of the strongest density of fibers crossing the NAc.

Image data sets, as well as “structures” autosegmented and drawn in Elements, were transferred to Gamma Plan (Elekta AB, Stockholm, Sweden). At the Gamma Plan treatment station (TPS), ACPC and a midpoint (MR) of the hemispheres were selected; often, this point corresponds to an area in the falx. Using the “Functional Target” option on the (TPS), the coordinates obtained in Elements were extrapolated to GK, and a dose of 90 Gy DMax was plotted with the corresponding isodose lines of 45 and 20 Gy, small adjustments were used to cover in a more complete visual sense the area that should correspond to the shell with the 20 Gy isodose line. Dose to the optic apparatus was recorded.

## Results

DTI connectivity analysis

We analyzed tractography studies from five subjects, designated as Patients 1 through 5, to ensure confidentiality. Patient 1 is a 34-year-old female, Patient 2 is a 46-year-old male, Patient 3 is an 83-year-old female, Patient 4 is a 63-year-old male, and Patient 5 is a 41-year-old male. None of the subjects exhibited structural neurological diseases.

In our DTI connectivity analysis of the NAc, we stratified the connections between the hippocampus, amygdala (AMYG), hypothalamus, different areas of the frontal lobes, ventral tegmental area (VTA), and middle structures of the thalamus to the NAc. The connections of these structures to the NAc, along with their fractional anisotropy (FA) values, are ordered from strongest to weakest in Table 1.

**Table 1:** NAc Connectivity analysis for 20,15, and 10 FA of cortical and subcortical structures involved in addiction’s neuronal pathways Table 1 represents structural connectivity between the nucleus accumbens and specific brain regions across three different fractional anisotropy thresholds: 20, 15, and 10. Patient number ¨1¨ represents the patient with the three Tesla MRI studies. Patients number 2,3,4, and 5 represent the 1.5-tesla MRI studies. The following algorithm was used for symbolic representation of high or low density connectivity:  0 = No density found, -  = weak density, + = average density,  +(+)= strong density. FA=fractional anisotropy, NAc= nucleus accumbens, AMYG= amygdala, dlFC= Dorsolateralfrontal cortex, mOFC= medial orbitofrontal cortex, lOFC= lateral orbitofrontal cortex, VTA= ventral tegmental area, PVG= periventricular gray

Connection	20 FA					15 FA					10 FA				
Patient #	1	2	3	4	5	1	2	3	4	5	1	2	3	4	5
mOFC NAc	+	0	+	+	0	+(+)	+(+)	+(+)	+(+)	+	+(+)	+(+)	+(+)	+(+)	+(+)
Hypothalamus NAc	+	0	0	0	0	+(+)	+(+)	+	+(+)	0	+(+)	+(+)	+(+)	+(+)	+
VTA NAc	0	0	0	+	0	0	+(+)	+	+(+)	0	0	+(+)	+(+)	+(+)	+
PVG	0	0	0	0	0	0	+	+	+	0	-	+(+)	+(+)	+(+)	+
AMYG NAc	0	0	0	0	0	0	+	0	+(+)	0	+	+	0	+(+)	+(+)
Insula-NAc	0	0	+	0	0	0	-	+(+)	-	0	+	+	+(+)	+	-
lOFC NAc	0	0	0	0	0	-	+(+)	0	0	-	0	+(+)	-	+	-
dlFC NAc	0	0	0	0	0	0	0	-	0	-	0	0	+	+	+
Hippocampus NAc	0	0	0	0	0	0	0	0	0	0	-	0	0	0	0

**Figure 1:**
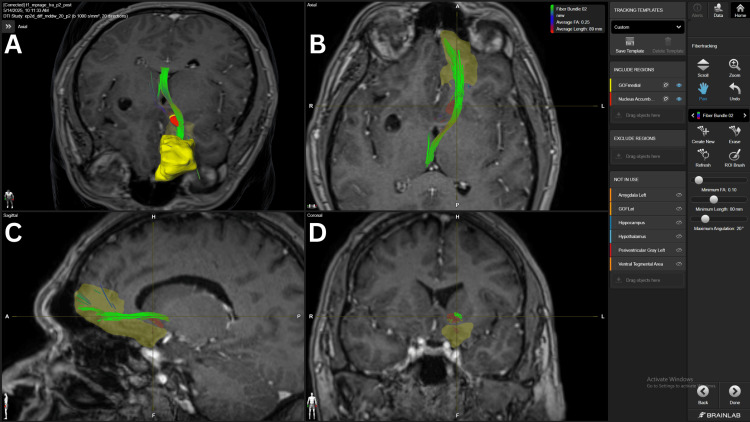
Fronto-accumbens fiber bundle in patient 5 Panel A. Three-dimensional reconstruction in an axial view, showing the green fiber bundle connecting the mOFC and NAc’s shell.  Panel B. Axial view of the fronto-accumbens fiber bundle crossing the superior and lateral aspects of the NAc. Panel C. Sagittal view of the fronto-accumbens fiber bundle with fibers reaching the medial and superior aspects of the mOFC.  Panel D. Coronal view of the fiber bundle crossing the superior and lateral aspects of the NAc. The yellow structure represents the mOFC and the red structure the NAc. mOFC= medial orbitofrontal cortex, NAc= nucleus accumbens BrainLab Elements (BrainLab, Munich, Germany) was used to generate the images.

The mOFC-NAc connection had the strongest connections found in our study. All patients, except for #5, presented a strong density fiber bundle (FB) at 15 FA. However, patient #5 did present a strong density FB with a 10 FA threshold. Three patients (#1, #3, #4) also presented with an average density FB at the 20 FA threshold. The fronto-accumbens connection is shown in Figure 1.

**Figure 2:**
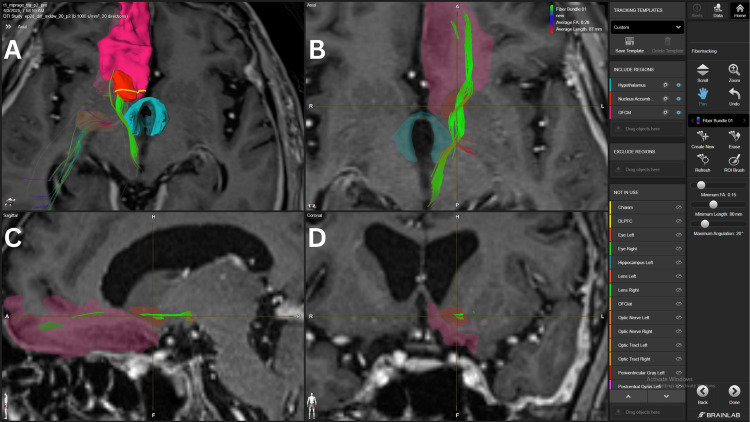
Fiber bundle connecting mOFC, NAc, and hypothalamus in patient 4 Panel A. Three-dimensional reconstruction in an axial view of the fiber bundle from the mOFC that crosses the NAc’s shell and ultimately the hypothalamus and medial structures in the brainstem. Panel B. Axial view of the fiber bundle crossing the lateral aspects of the mOFC, NAc, and hypothalamus. Some mixed red fibers are also visualized, probably involving the uncinate fasciculus. Panel C. Sagittal view of the fiber bundle crossing the lateral and inferior aspects of the NAc. Panel D. Coronal view of the green fiber bundle crossing the lateral aspect of the NAc. The pink structure corresponds to the mOFC, the red structure to the NAc, and the light blue structure to the hypothalamus. mOFC=medial orbitofrontal cortex, NAc= nucleus accumbens BrainLab Elements (BrainLab, Munich, Germany) was used to generate the images.

The hypothalamus-NAc connection exhibited the second strongest association fiber bundles, with a strong density found in four patients with 10 (#1, #2, #3, #4) and three with 15 (#1, #2, #4) FA thresholds. Also, we found three patients with average density FB with 10 (#5), 15 (#3), and 20 (#1) FA thresholds. A fiber bundle coming from the mOFC, crossing the NAc’s shell, and reaching the lateral hypothalamus is shown in Figure 2. 

**Figure 3:**
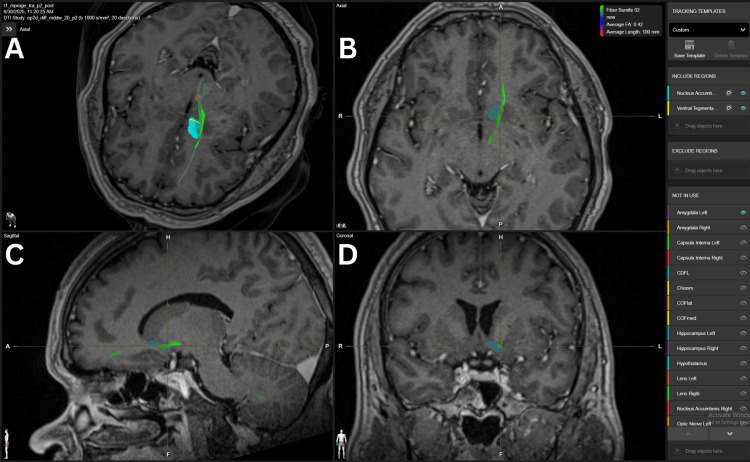
NAc’s connection to VTA in patient 2. Panel A. Three-dimensional reconstruction of the MFB connecting the NAc-VTA.  The yellow structure represents the VTA. Panel B. Axial view of the green fiber bundle crossing the lateral aspect of the NAc. Panel C. Sagittal view of the green fibers crossing the lateral aspect of the NAC. Panel D.  Coronal view showing the green fibers crossing the NAc in its lateral aspect. Light blue structures represent the NAc. VTA= ventral tegmental area, NAc=nucleus accumbens, MFB=medial forebrain bundle BrainLab Elements (BrainLab, Munich, Germany) was used to generate the images.

The VTA-NAc connection presented a strong density FB in two patients (#2, #4) with 15 FA and in three patients (#2, #3, #4) with 10 FA. Also, average density FB was identified in three patients with 20 (#4), 15 (#3), and 10 (#5) FA. The VTA-NAc connection is illustrated in Figure 3. 

**Figure 4:**
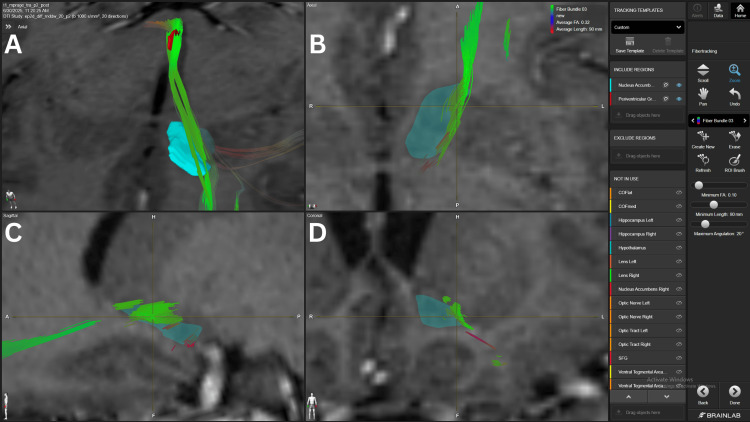
NAc-PVG connection in patient 2 Panel A. Three-dimensional reconstruction of the PVG-NAc connection, in an axial view. Fiber bundle reaching the lateral aspect of the  NAc coming from the dorsomedial structures of the thalamus, represented by the PVG, shown in the red structure. Panel B. Axial view of the fiber bundle crossing the lateral aspect of the NAc. Panel C. Sagittal view of the fiber bundle crossing the superior aspect of the NAc. Panel D. Coronal view of the fiber bundle crossing the lateral aspect of the NAc. The light blue structure represents the NAc. NAc=nucleus accumbens PVG= periventricular grey BrainLab Elements (BrainLab, Munich, Germany) was used to generate the images.

The PVG with a 4.0 mm margin was used as an anatomical representation of the medial structures of the thalamus and its connection to the NAc. This connection showed a strong density FB with 10 FA in three patients (#2, #3, #4) and an average density FB in the same patients with 15 FA and in one patient (#5) with 10 FA, as shown in Figure 4.

**Figure 5:**
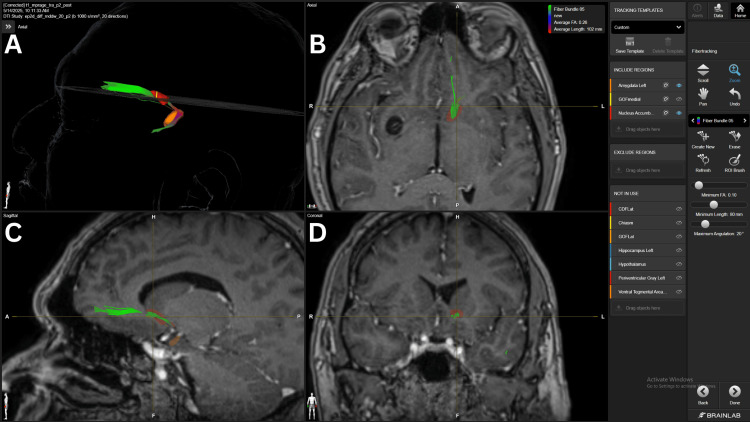
Fiber bundle connecting the mOFC-NAc-AMYG in patient 5 Panel A. Three-dimensional reconstruction of the green fiber bundle connection coming from the mOFC and then crossing the shell of the NAc, finally reaching the AMYG. Panel B.  Axial view of the fiber bundle crossing the central region of the NAc. Panel C. Sagittal view of the fiber bundle coming from the mOFC, crossing the NAc, and turning inferiorly seeking the AMYG. Panel D. Coronal view of the fiber bundle crossing the inferior aspect of the NAc. The mOFC structure was removed to visualize the fiber bundle coming from the prefrontal structure. mOFC= medial orbitofrontal cortex, NAc= nucleus accumbens, AMYG=amygdala BrainLab Elements (BrainLab, Munich, Germany) was used to generate the images.

Also, a weak density FB in one patient (#1) with 10 FA. The AMYG-NAc connection was found to have a strong density FB with the 10 in two patients (#4, #5) and in one patient with 15 (#4) FA thresholds. Average density FB was found with 10 FA in two patients (#1, #2) and with 15 FA in one patient (#2). A specific FB connecting the mOFC-NAc-AMYG was also found, as shown in Figure 5.

**Figure 6:**
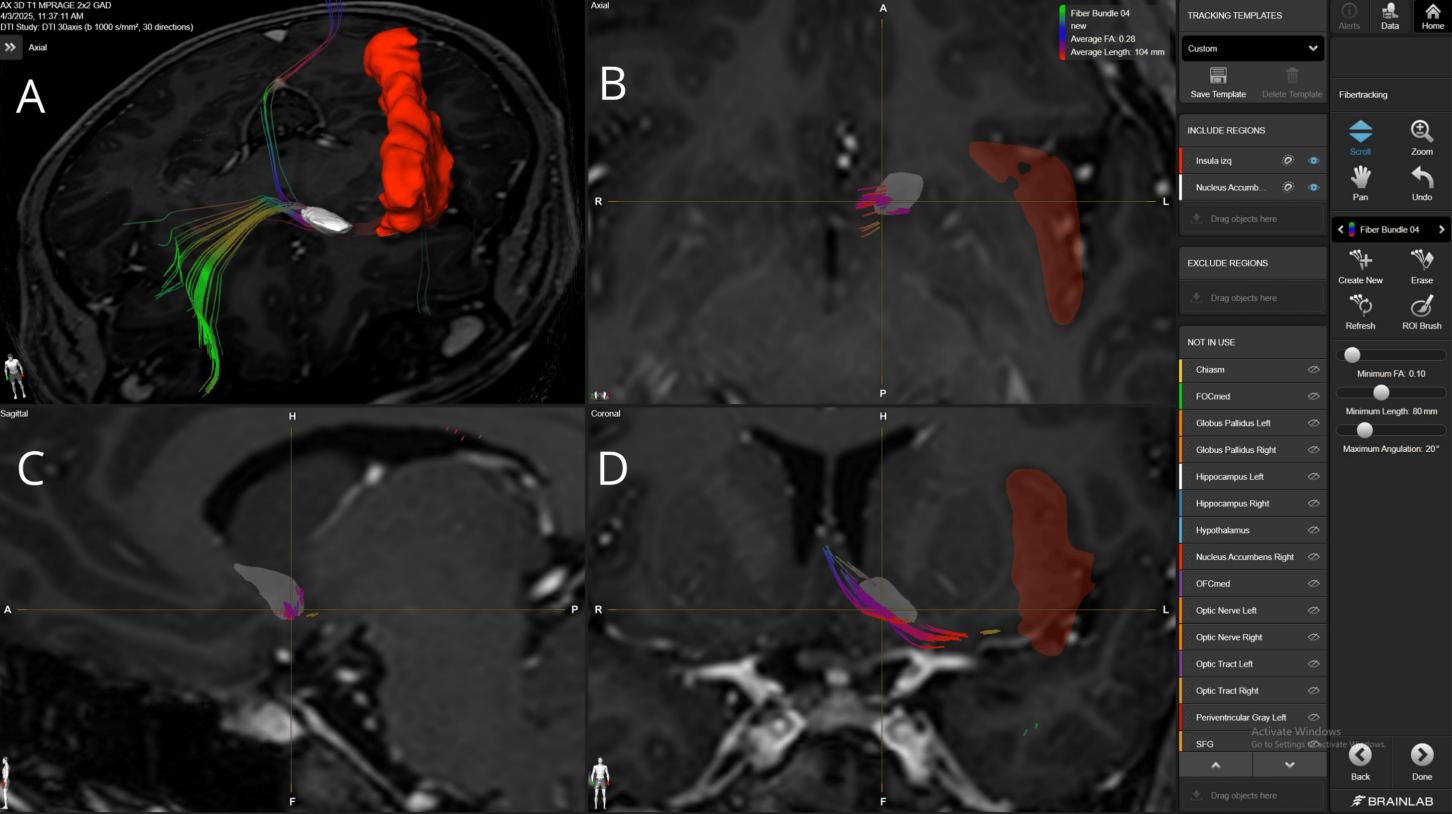
Connection between the insular cortex and the shell of the NAc in patient 1 Panel A. Three-dimensional reconstruction of the fiber bundle connecting the insula with the NAc in an axial view. The red structure represents the insular cortex, while the white structure represents the NAc. Additionally, a green fiber bundle originating from the contralateral mOFC is observed to extend to the NAc and Insula. Panel B. Axial view of the fiber bundle crossing the medial and posterior aspect of the NAc. Panel C. Sagittal view of the fiber bundle crossing the ventral aspect of the NAC. Panel D. Coronal view of the fiber bundle crossing the medial and ventral aspect of the NAc reaching the caudal aspect of the insula. NAc=nucleus accumbens, mOFC medial orbitofrontal cortex BrainLab Elements (BrainLab, Munich, Germany) was used to generate the images.

The Insula-NAc connection presented strong density fiber bundles in one patient (#3) with 15 and 10 FA thresholds. Additionally, an average density of fiber bundles was observed in one patient (#3) with 20 FA and in three patients (#1, #2, #4) with 10 FA. Weak connections were found in two patients (#2, #4) with 15 FA, and in one patient (#5) with 10 FA. The insula-NAc connection is illustrated in Figure 6.

**Figure 7:**
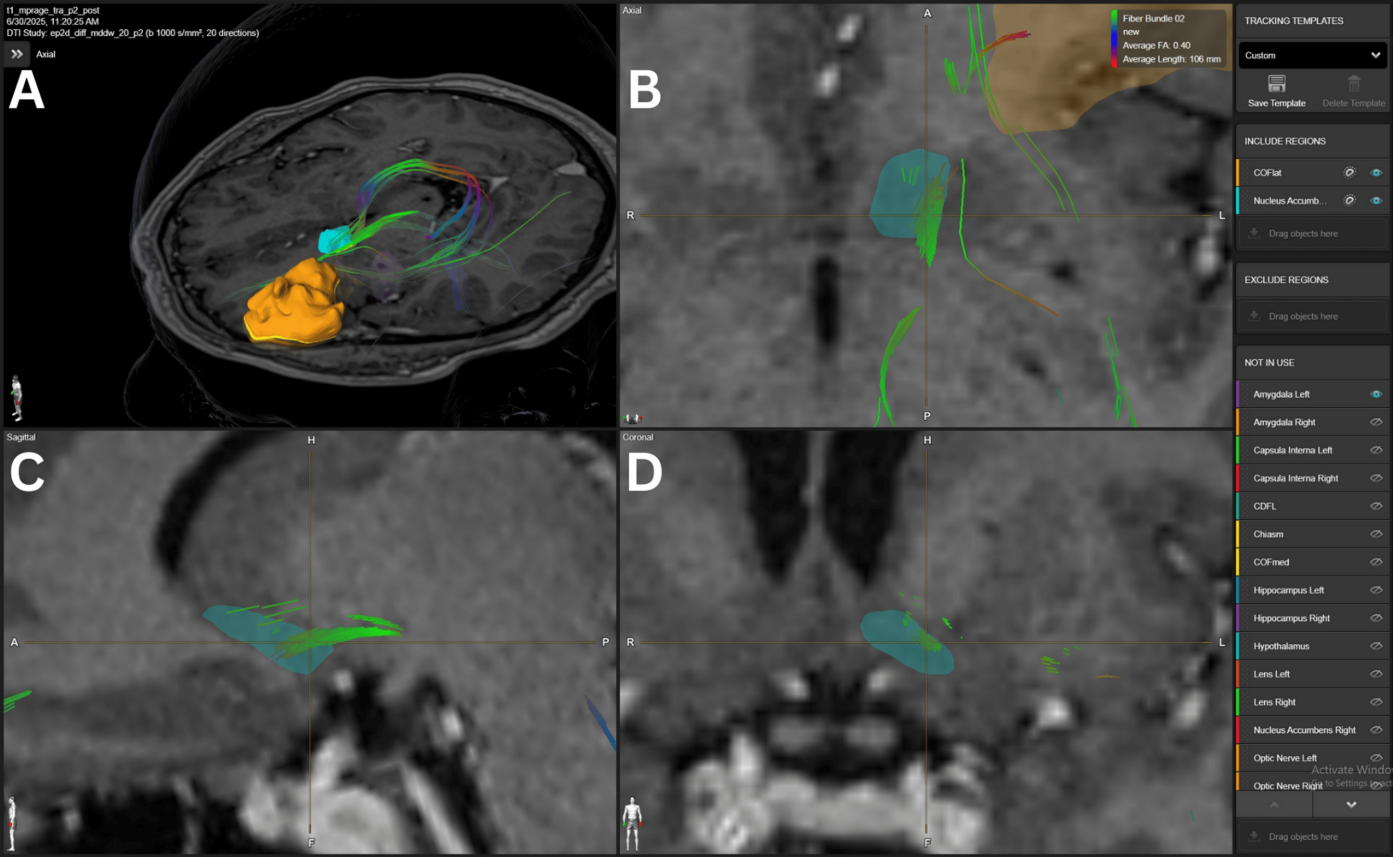
Connection between the lOFC and NAc in patient 2 Panel A. Three-dimensional reconstruction of the green fiber bundle connection of the lOFC (orange) with the superior and lateral aspect of the NAc (light blue) in an axial view. Panel B. Axial view of the fiber bundle crossing the lateral aspect of the NAc. Panel C. Sagittal view of the fiber bundle crossing the inferior aspect of the NAC. Panel D. Coronal view of the fiber bundle crossing the lateral and superior aspect of the NAc. lOFC= lateral orbitofrontal cortex, NAc=nucleus accumbens BrainLab Elements (BrainLab, Munich, Germany) was used to generate the images.

A marked difference between prefrontal structures was evident, especially when comparing mOFC to lOFC and dlFC. The lOFC-NAc connection presented strong density FB with 15 and 10 FA in patient 2, and average density FB in patient 4 with 10 FA; the rest of the connections were all weak density FB with 10 (#3, #5) and 15 (#1, #5). The dlFC-NAc connection was found to have only average density FB with the 10 FA in three patients (#3, #4, #5), and a weak density FB with the 15 FA in two patients (#3, #5). The lOFC connection is shown in Figure 7.

**Figure 8:**
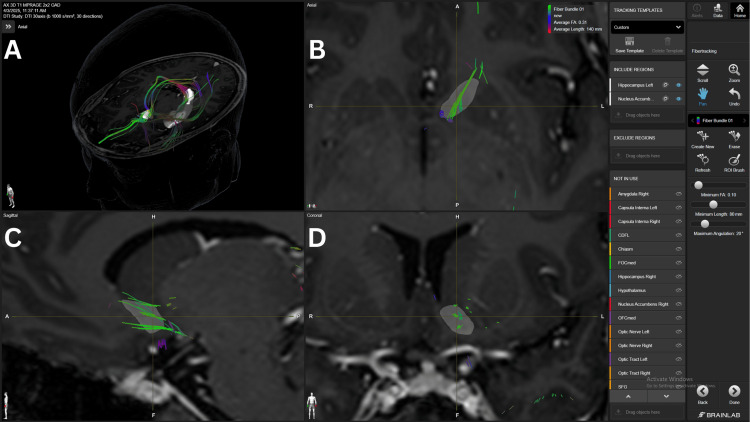
NAc’s connection to hippocampus in a 3-Tesla DTI study in patient 1 Panel A. Three-dimensional reconstruction of the green fibers crossing the NAc and traveling through the fornix from the hippocampus. The white upper, anterior, and smaller structure represents the NAc, while the lower, posterior, and bigger structure represents the hippocampus. Panel B. Axial view of the fiber bundle crossing the NAC from the lateral to medial aspect. Panel C.  Sagittal view of the fiber bundle crossing the central region of the NAc. Panel D. Coronal view of the fibers crossing the central regions of the NAc. NAc= nucleus accumbens, DTI=diffusion tensor imaging BrainLab Elements (BrainLab, Munich, Germany) was used to generate the images.

The Hippocampus-NAc had a minor connection in our analysis, with only one connection found in the three-Tesla images, with a weak density fiber in the 10 FA thresholds, as illustrated in Figure 8.

**Figure 9:**
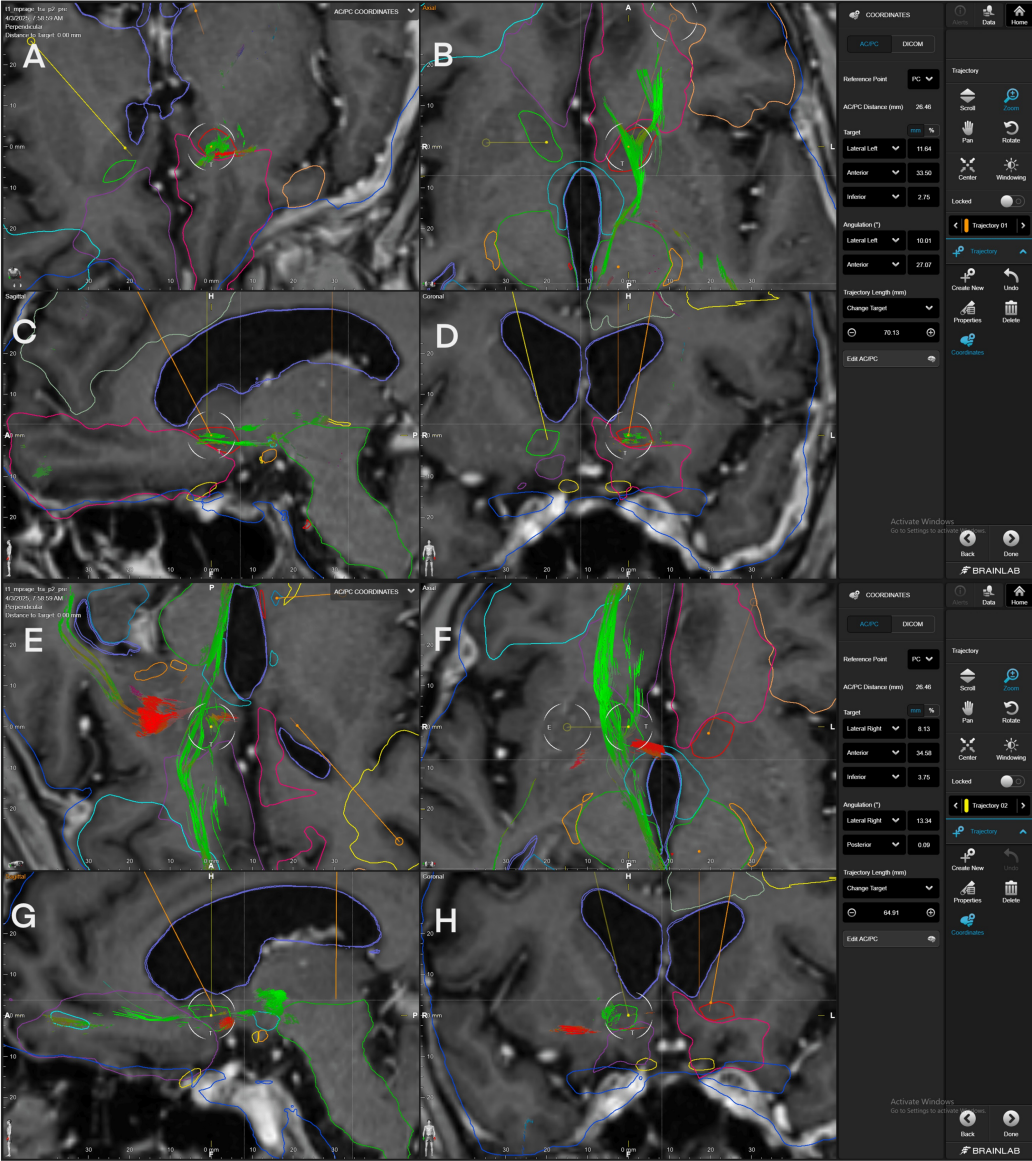
Left and right trajectories pointing to the strongest-density fibers passing through the NAc in patient 4 Panel A. Superior view of the left target trajectory to the strongest density fiber bundle. Panel B. Axial view of the strongest density left fiber bundle crossing the NAc. Panel C. Sagittal view of the strongest density left fiber bundle crossing the NAc. Panel D. Coronal view of the strongest density left fiber bundle crossing the NAc. Panel E. Superior view of the right target trajectory to the strongest density fiber bundle. Panel F. Axial view of the strongest density right fiber bundle crossing the NAc. Panel G. Sagittal view of the strongest density right fiber bundle crossing the NAc. Panel H. Coronal view of the strongest density right fiber bundle crossing the NAc. A strong-density fiber bundle is identified crossing the left and right ventromedial NAc, particularly the shell of the NAc. These fibers involve the connections of the left  (panel B-D) and right (panel F-H) mOFC-NAc, AMYG-NAc, hypothalamus-NAc, VTA-NAc, and PVG-NAc from each hemisphere. The fibers travel in the axial plane, crossing the mOFC, NAC, and hypothalamus, reaching the medial thalamus. mOFC=medial orbitofrontal cortex, AMYG= amygdala, VTA= ventral tegmental area, PVG= periventricular gray, NAc= nucleus accumbens BrainLab Elements (BrainLab, Munich, Germany) was used to generate the images.

The five most densely connected fibers to the NAc were selected from a 1.5-tesla study and then activated simultaneously to visually create a single fiber bundle, which was used for target selection as shown in Figure 9.

Once the connections were established in the stereotactic module of the Elements program, the images were aligned to the anterior and posterior commissure (ACPC), and a target was selected for each hemisphere at the highest density of fibers crossing the NAc. Stereotactic coordinates for targeting obtained from the visual assessment are depicted in Table 2.

**Table 2:** Coordinates from the connectomic study Coordinates were obtained according to the ACPC line, for each hemisphere and their fiber bundle and structures. ACPC= anterior and posterior commissure, PC= posterior commissure

COORDINATES	RIGHT	LEFT
Lateral	8.1	11.6
Anterior TO PC	34.6	33.5
Inferior	3.7	2.7

Specific regions of interest (ROIs) connected to the NAc for target selection included the mOFC, AMYG, hypothalamus, VTA, and PVG, and their specific FA thresholds are described in Table 3.

**Table 3:** Right and left connections to NAc for target selection and FA thresholds Table 3 represents the individual connections to NAc for each hemisphere and the respective FA used. The activation of all fibers allowed for visual assessment to make target selection in the higher point of fiber density crossing the NAc. NAc= Nucleus accumbens, FA= fractional anisotropy, mOFC= Medial OrbitoFrontal Cortex, AMYG= Amygdala, VTA= Ventral Tegmental Area, PVG= Periventricular Gray.

Right Fibers			Left Fibers		
Structures	# Fiber Bundle	FA	Structures	# Fiber Bundle	FA
NAc-mOFC	6	16	NAc-mOFC	1	20
NAc-AMYG	7	10	NAc-AMYG	2	15
NAc-Hypothalamus	8	11	NAc-Hypothalamus	3	15
NAc-VTA	9	10	NAc-VTA	4	20
NAc-PVG	10	15	NAc-PVG	5	15

**Figure 10:**
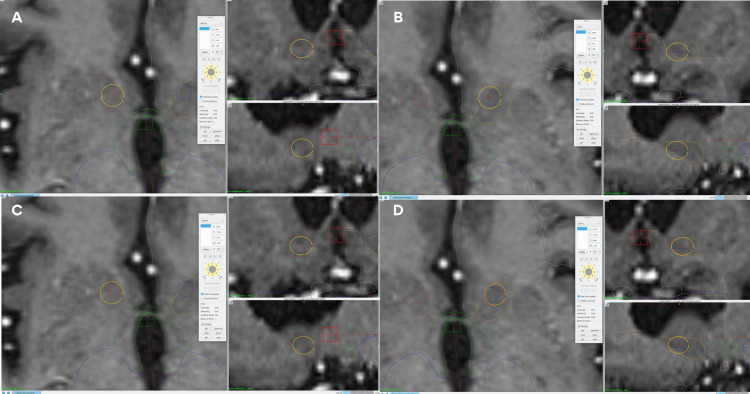
Original target coordinates vs refined target in patient 4 Panel A. Axial, sagittal, and coronal views of the right shot with the original coordinates. Panel B. Axial, sagittal, and coronal views of the left shot with the original coordinates. Panel C. Axial, sagittal, and coronal views of the right refined shot with a slight lateralization from the original coordinates. Panel D. Axial, sagittal, and coronal views of the left refined shot with a slight lateralization from the original coordinates. The yellow circle represents the 50% isodose line in all panels and views. In panels A and C, the red line represents the right NAc. In panels B and D, the orange line represents the left NAc.

The exact coordinates extracted from Brainlab Elements (BrainLab, Munich, Germany) were plotted on the TPS, using the “Functional Target” option. However, a slight lateralization of the two targets was made to refine the shot, predominantly, to the shell, as shown in Figure 10.

**Figure 11:**
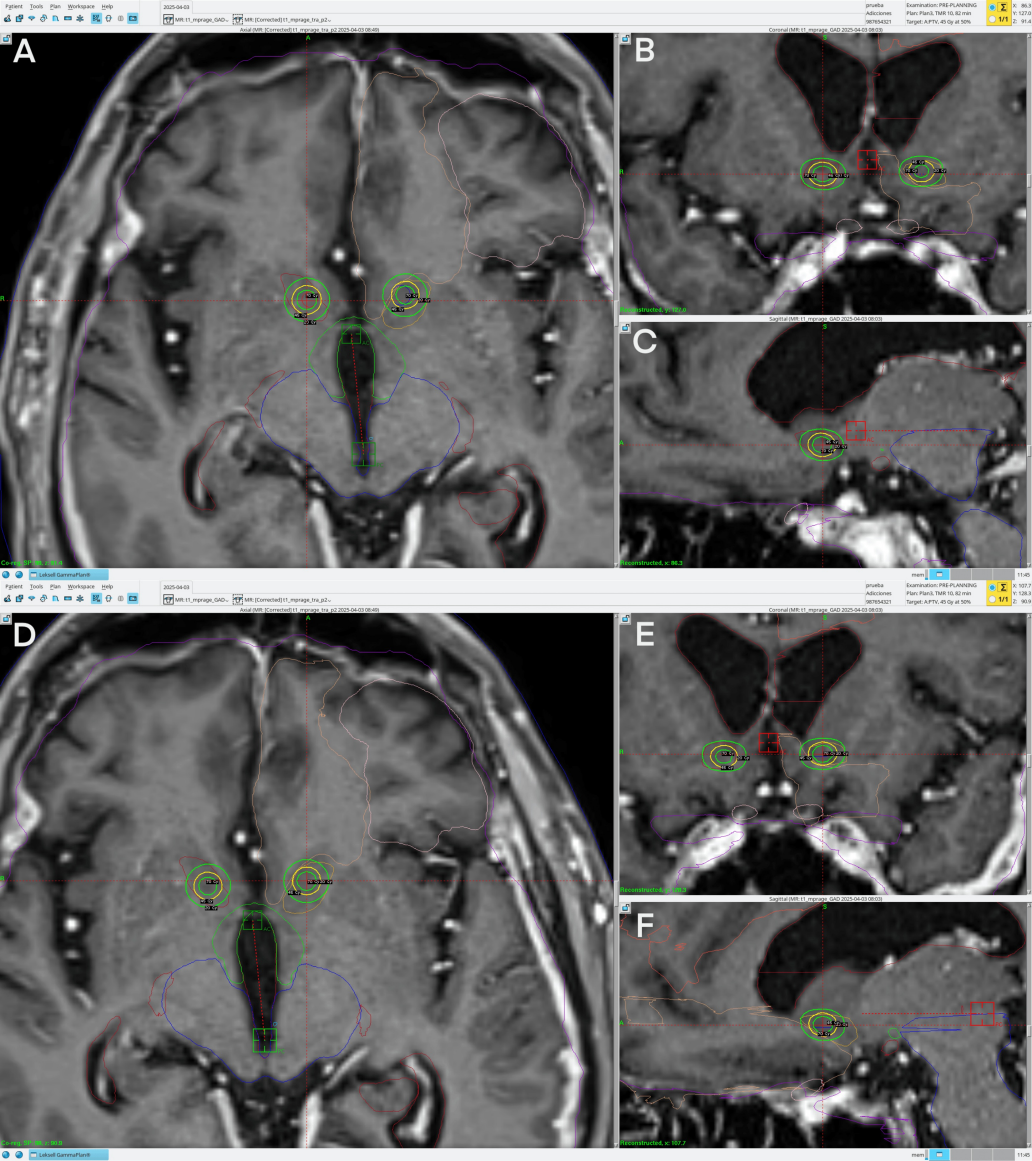
Treatment planning on Gamma Knife for patient 4 Panel A. Axial view of the right NAc shot. Panel B. Coronal view of the right NAc shot. Panel C. Sagittal view of the right NAc shot. Panel D. Axial view of the left NAc shot. Panel E. Coronal view of the left NAc shot. Panel F. Sagittal view of the left NAc shot. Each panel has an inner green line corresponding to the 70 Gy isodose line, a yellow line corresponding to the 45 Gy isodose line, and an outer green isodose line corresponding to the 20 Gy isodose line. NAc= nucleus accumbens

A prescription dose of 45 Gy to the 50% isodose line with a 4.0 mm collimator shot to the NAc, bilaterally. The shot was adjusted, as mentioned above, to better align the 20 Gy isodose line with the NAc volume, looking for a potential neuromodulation dose/effect (non-necrotic) in this zone, as shown in Figure 11.

Doses and their proportions to NAc volumes were extracted from TPS, and are as follows: the right NAc total volume was 0.316 cc. 0.238 (75.2%) of the right NAc was receiving> 20 Gy. The mean dose in Gy was 36.7, and Dmax was 90 Gy. The left NAc total volume was 0.381 cc. 0.212 (55.6%) of the left NAc was receiving> 20 Gy. The mean dose in Gy was 30.7, and Dmax was 90 Gy. In our treatment plan proposal, the highest doses for organs at risk (OAR) were received by the chiasm and right optic tract, with both receiving 5.4 Gy as Dmax; the rest of the optic pathway was below this dose.

## Discussion

Functional anatomy of the NAc: implications in addiction treatment

**Figure 12:**
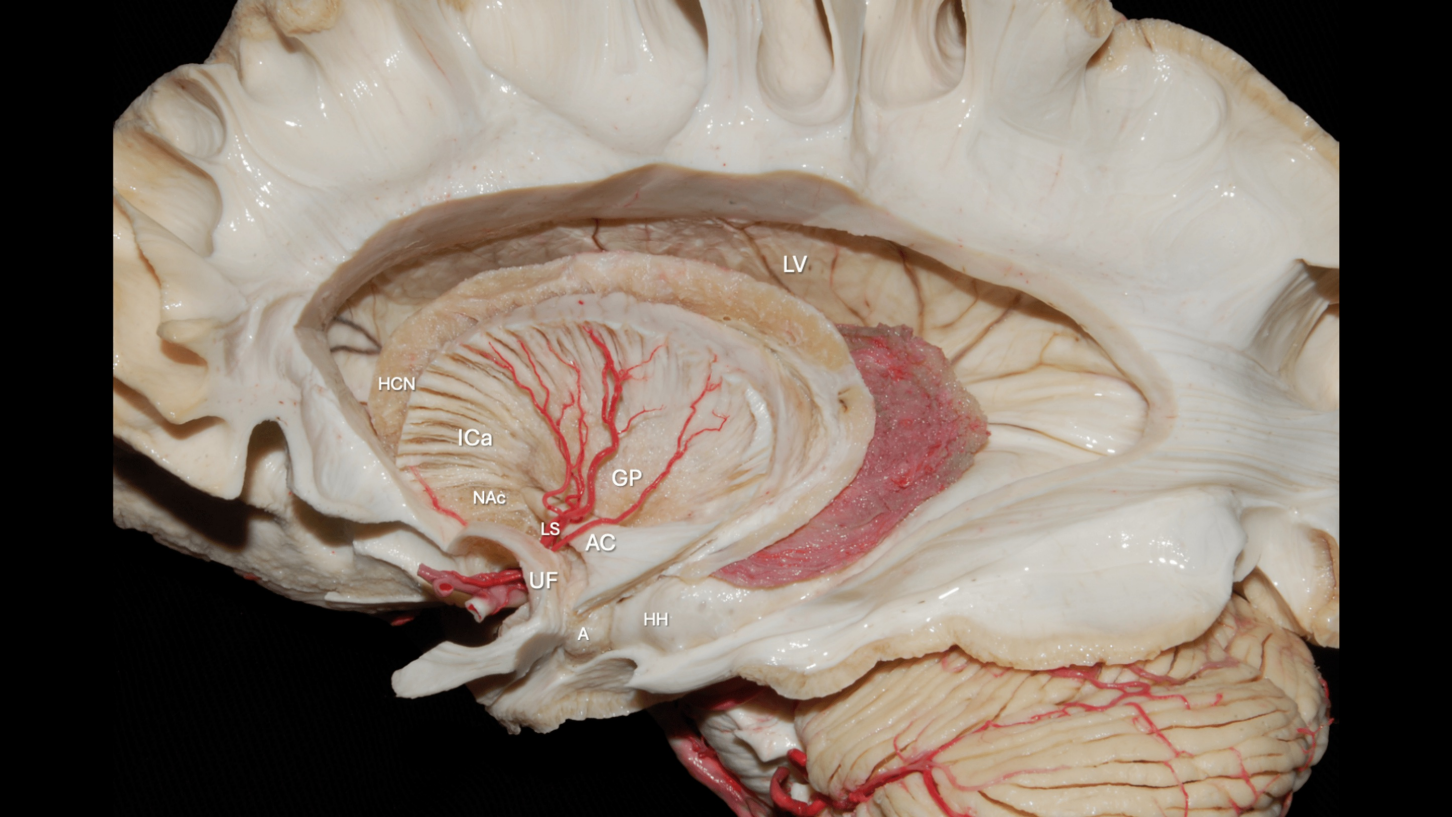
Dissection of the lateral surface of the left cerebral hemisphere showing the central core region The Nucleus Accumbens (NAc) is related to the Anterior Commissure (AC), Uncinate Fasciculus (UF), anterior genu of the Internal Capsule (ICa), head of the Caudate Nucleus (HCN), Amygdala (A), and head of the Hippocampus (HH). The course of the lenticulostriate arteries in relation to these structures is also demonstrated. The Putamen has been resected, exposing the Globus Pallidus (GP) in depth. Original anatomical white matter dissection performed by author R.G. Parraga and edited by author J.E. Chang.

**Figure 13:**
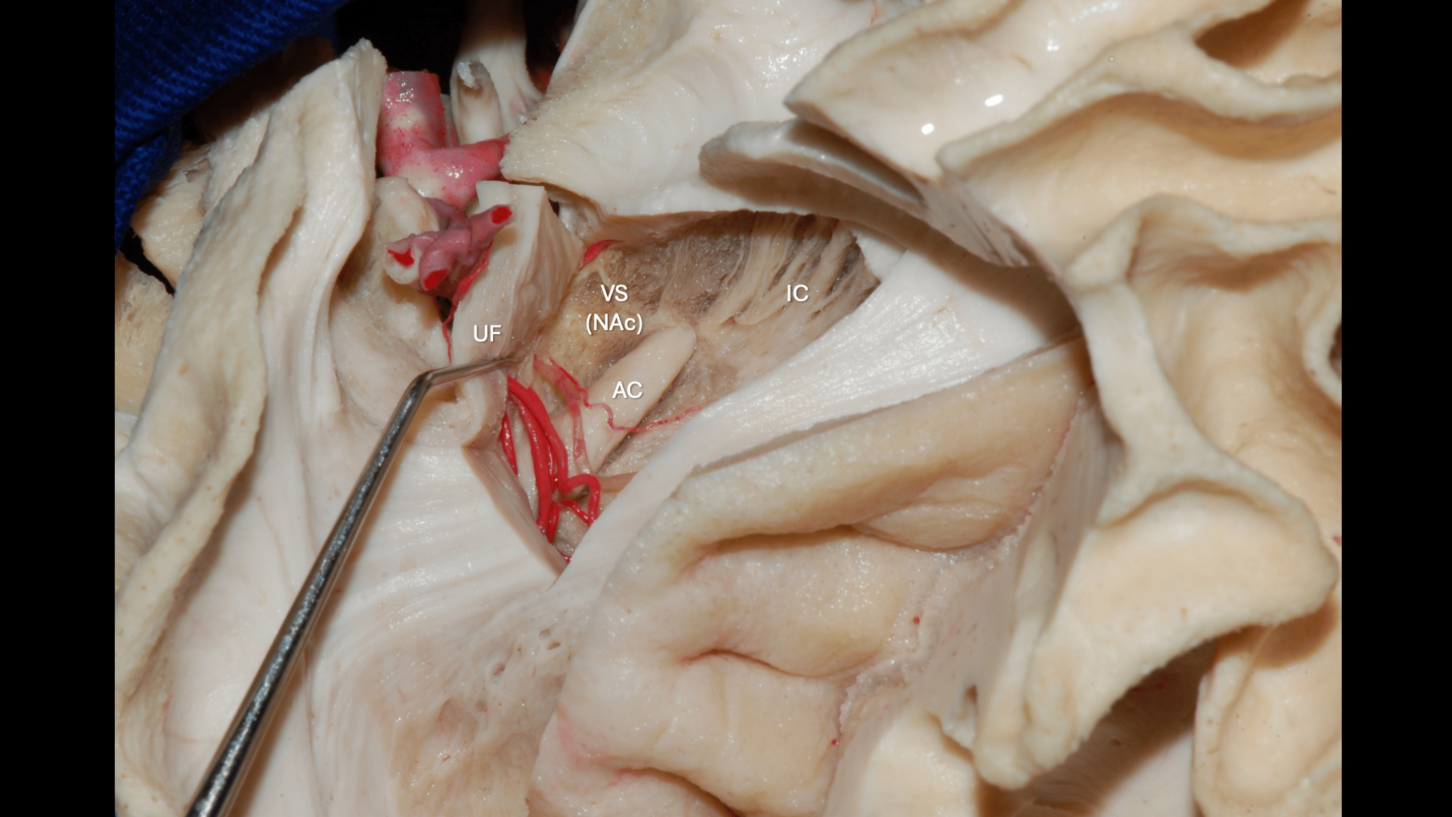
Superior view of the Nucleus Accumbens Superior view showing the Nucleus Accumbens (NAc), the main component of the ventral striatum, located anterior to the Anterior Commissure (AC), inferior to the anterior genu of the Internal Capsule (ICa), and medial-superior to the Uncinate Fasciculus (UF). Original anatomical white matter dissection performed by author R.G. Parraga and edited by author J.E. Chang.

The NAc is a central structure in the brain’s reward and motivation system, integrating inputs from emotional, cognitive, and motor regions to guide goal-directed behavior. Originally termed nucleus accumbens septi for its position “lying against” the septum (accumbere, Latin: “to recline”), it forms the main component of the ventral striatum, situated rostral to the anterior commissure, below the anterior limb of the internal capsule, and adjacent to the caudate nucleus and putamen, as shown in Figures 12, 13.

This location allows the NAc to link limbic and basal ganglia circuits, underlying its role in addiction [8,9]. The NAc consists of two subregions: core and shell. The core is central and lateral, resembling the dorsal striatum, and mediates learned action-outcome associations and motor planning. The shell surrounds the core medially, laterally, and ventrally, with a heterogeneous structure and strong limbic connections, supporting reward salience, emotional processing, and motivational evaluation [9-11].

Glutamatergic input arises from the hippocampus, basolateral amygdala, and prefrontal areas, including the mPFC, OFC, ACC, and insula [12,13]. These pathways convey information about context, memory, and expectation, shaping reward-guided behaviors. Prefrontal fibers traverse the anterior limb of the internal capsule, while hippocampal fibers travel via the fornix, primarily targeting the shell [14], as shown in Figures 2, 8. Amygdala projections follow the ventral amygdalofugal pathway, integrating emotional significance [12,14]. Dopaminergic input from the VTA via the medial forebrain bundle (Figure 3) modulates reinforcement, with the shell showing higher receptor density for D1 and D2, enhancing reward evaluation and attention to salient stimuli [15].

Efferent projections mainly target the ventral pallidum, with core outputs influencing basal ganglia loops through the dorsolateral VP, and shell outputs reaching ventromedial VP, hypothalamus, and brainstem structures, including the pedunculopontine tegmental nucleus [16,17]. Bidirectional connections with the lateral hypothalamus (Figure 2) and VTA regulate dopamine signaling and autonomic responses.

DTI and high-angular resolution diffusion imaging (HARDI) imaging reveal consistent fiber bundles, such as fronto-accumbens tracts (Figure 1) from the mPFC and ACC, hippocampal fibers along the fornix (Figure 8), and curved amygdala pathways near the external capsule, confirming classical anatomical studies and mapping detailed NAc subregions [18,19]. Notably, medial shell connections to the subgenual cingulate (Brodmann area 25) and ventromedial prefrontal cortex are implicated in mood regulation and reward dysfunction in addiction. Despite these well-known neuroanatomical connections, they were inconsistent throughout the five subjects studied with Elements. However, connections could be further intensified by lowering the FA below 10, which provided a weak or unreliable image for targeting, exemplifying one of the current shortcomings of the current version of Elements used in this technical description.

These neuroanatomic features contribute to the NAc’s vulnerability to addictive behaviors. Chronic drug exposure induces lasting neuroplastic changes, including enhanced dopamine sensitivity in the shell and reduced prefrontal control, promoting compulsive reward-seeking [20,21]. Thus, a more detailed visualization of the connections through commercially available software could furthermore assist in refining a potential target and serve to establish common guidelines and effectiveness, comparison parameters for targeting the NAc in the near future for the treatment of addictions.

The NAc has been investigated as a surgical target for addiction treatment for several decades, with outcomes that have varied across studies. Early interventions using DBS or RF ablation demonstrated that modulating or destroying this region could reduce relapse rates, although the efficacy was inconsistent and depended on patient selection, target localization, and surgical methodology [5]. These findings underscore both the potential and the challenges of targeting the NAc in addictive disorders.

Previously, our group published a systematic review of surgical interventions, either DBS or RF ablation, to the NAc, which overall showed promising results. Overall treatment response, as categorized as non-relapses for opioids, alcohol, and nicotine, for both DBS and RF thermal lesion, hovered between 50 and 75% [5]. Also, medication-assisted treatment (MAT), in combination with behavioral therapy, is considered the gold standard treatment for patients with OUD [22]. MAT with medications like buprenorphine plus medical counseling for OUD has been reported to decrease the mean baseline rate per week. However, only 43% of patients had abstinence success with this treatment [23], potentially favoring neurosurgical interventions in treatment success, or even better, the potential synergistic effect of neurosurgical interventions on the NAc and MAT.

Recent DBS studies targeting the NAc for refractory substance use disorders, and specifically, in treatment-resistant OUD, preliminary trials [24] report that NAc/ventral capsule DBS is safe and well tolerated, with no serious device-related adverse events. Sustained opioid abstinence (>500 days) was achieved in half of the patients, alongside reductions in craving, depression, and anxiety. In alcohol use disorder, a double-blind RCT [6] demonstrated non-significant differences in total abstinence between DBS and sham groups (n=12), but secondary analyses revealed significant improvements in craving, anhedonia, and abstinent days in the active DBS group. In a long-term open-label study from China [25], simultaneous stimulation of the NAc and ALIC in heroin-dependent individuals yielded >three years of abstinence in five of eight participants, reduced craving severity (p< 0.001), and increased glucose metabolism in prefrontal areas.

A study investigating the validity and reliability of NAc projections reconstructions based on probabilistic tractography in 11 healthy subjects who underwent 3T MRI studies at seven different times, found NAc connectivity to: AMYG, anterior cingulate cortex (ACC), medial thalamus, Hippocampus, mPFC, and VTA [26]. This was consistent with our study except for the hippocampus, which had only very low-density connections to NAc with 10 FA in the 3T patient, and ACC, which was not evaluated in our study, representing another potential shortcoming of this study and its methodology. 

They also perform a literature analysis of the NAc afferent and efferent connectivity in animals, and report that the most frequently described connections were from NAc to Thalamus with 151, followed by the AMYG with 79, VTA with 56, hippocampus with 54, Hypothalamus with 54, substantia nigra 36, prefrontal cortex 30, and lastly the ventrales pallidum with 26 [26]. This corresponds closely with our findings, demonstrating connectivity in the following descending order: MOFC, HYPO, VTA, PVG, and AMYG.

As described before, the NAc shell receives the majority of dopamine inputs, and dopamine levels are also higher than in the core. The Medium spiny neurons (MSNs) also differ between NAc. MSNs in the shell have lower action potential thresholds and consequently a greater and faster action potential and firing frequency, respectively [27]. Lastly, and well-documented, the shell has also been closely associated with the reward and motivational system. Therefore, the rationale for targeting, predominantly, the Shell of the NAc for addiction treatment.

A recent study demonstrated the feasibility of linear accelerator-based radiosurgery targeting the NAc for addiction treatment [28]. The study proposed three different radiosurgical approaches, each delivering ablative doses between 120 and 140 Gy to the NAc bilaterally. Even though they proposed higher radiation doses and two, four, and six-shot approaches, the only plan not feasible was the six-shot approach, which exceeded the 10 Gy constraint for the chiasm in a single fraction at their institution. Their results, alongside our study, demonstrate that stereotactic radiosurgery (SRS) to NAc for addiction may be feasible across different radiosurgery technologies.

Even though the above study proposes an ablative approach with higher doses and multiple shots, it is less than the 90 Gy proposed by our group. As seen in one of our patients treated for intractable pain and comorbid anxious-depressive disorder, in which we performed a single-shot irradiation to the anterior cingulum bundle (CB) bilaterally, with 90 Gy as Dmax, to modulate the neuronal circuit rather than to disrupt the fibers. However, over time (eight months for left CB and 12 months for right CB), the 90 Gy dose proved to be ablative, and a classical image of cingulotomy was evident on the DTI study at 12-month follow-up [29]. In another publication by our group, it was clinically demonstrated that 11 patients with oncological pain were treated with a triple-target approach (hypophysis + medial thalamus), using a single 90 Gy dose, and proved to be effective, achieving pain reduction (>50%) in 63.63% of cases (n=7). For this group, the pain score on the Numerical Rating Scale (NRS) decreased from a pre-treatment median of 10 to five post-treatment, with a radiomodulation effect evidenced in seven patients, and a median time to response of 2.5 days [30]. These results suggest that 90 Gy as Dmax may suffice in modulating and possibly altering a neuronal circuitry permanently by means of necrotic effect.

Limitations

This study is limited by two main factors: a small sample size of healthy subjects, with no addicted patients included in this cohort, and reliance on subjective visual estimation of fiber density. Lastly, a major limitation to the process described here for connectomic-based radiosurgery is that it relies on a subjective, visual estimation of fiber density, which could be highly variable between interoperators trying to achieve the same objective.

Future directions

The inclusion of a larger sample size of addicted patients for a connectomic-based radiosurgery study is needed in the future. Also, the use of automated software-based estimation of fiber density may abolish visual estimation bias and inter-examiner variability in the near future. Lastly and most critically, the safety and efficacy of radiosurgical treatment for addiction remain theoretical, necessitating a future clinical trial with the proper establishment of eligibility criteria, treatment specification, and follow-up to validate these outcomes.

## Conclusions

Addiction is a prevalent disease and the cause of a holistic burden in society, which, despite medical, behavioral, and invasive treatment, still has high relapse rates over time that need to be addressed. Radiosurgery is a reemerging treatment modality for functional indications that has been time-proven, especially in its safety profiles, and may offer a novel treatment option for addiction. Connectivity-based radiosurgery, using commercially available software, proved useful for visualizing most NAc connections, although not all, and for autosegmenting the NAc, which might enhance targeting planning for patient/disease-specific treatments.
